# *PGA37* Overexpression Promotes Chloroplast Development in *Arabidopsis* Roots Through Direct Transcriptional Activation of *GLK2*, *ARR13*, and *ARR21*

**DOI:** 10.3390/plants14091270

**Published:** 2025-04-22

**Authors:** Yunfeng Wei, Huiping Yang, Yujing Wang, Huimin Shen, Shuwei Zhang, Zhirong Yang, Ling Yuan, Xingchun Wang

**Affiliations:** 1 Houji Laboratory in Shanxi Province, College of Life Sciences, Shanxi Agricultural University, Taigu, Jinzhong 030801, China; weiyf_hero@163.com (Y.W.); ffy18137020725@163.com (H.Y.); wangyujing0420@163.com (Y.W.); 2 College of Agriculture, Shanxi Agricultural University, Taiyuan 030031, China; shenhuimin961210@163.com (H.S.); zshuwei@sxau.edu.cn (S.Z.); 3 Department of Plant and Soil Sciences, Kentucky Tobacco Research and Development Center, University of Kentucky, Lexington, KY 40546, USA; 4 Department of Basic Sciences, Shanxi Agricultural University, Taigu, Jinzhong 030801, China

**Keywords:** Arabidopsis, chloroplast biogenesis, transcription factor, PGA37, GLK2, ARR13, ARR21, cytokinin signaling

## Abstract

Chloroplast biogenesis and development are essential processes in plants, profoundly influencing their growth, survival, and productivity. However, the transcription factors controlling chloroplast development, especially in roots, are poorly characterized. Here, we demonstrate that the ectopic expression of the seed-specific transcription factor Plant Growth Regulator 37 (PGA37) promotes chloroplast development in roots, causing root-greening. Using a steroid-inducible gene expression system and RNA-Seq, we identified 97 potential PGA37 target genes. Notably, PGA37 directly activates the transcription factor GOLDEN2-LIKE (GLK2), which governs chloroplast biogenesis. An overexpression of *GLK2* replicated the root-greening phenotype observed in PGA37-overexpressing plants, while GLK2 mutation significantly reduced chlorophyll content and suppressed root-greening in PGA37-overexpressing seedlings. Furthermore, PGA37 directly binds to the promoters of type-B response regulators ARR13 and ARR21, thereby activating the cytokinin signaling pathway. Mutations in these regulators partially diminished chlorophyll accumulation in PGA37-overexpressing seedlings, suggesting that PGA37-regulated chloroplast development is partially mediated by the cytokinin signaling through ARR13 and ARR21. Taken together, we propose that PGA37 orchestrates chloroplast development by coordinately regulating transcription factors from various families, including GLK2, ARR13, and ARR21, positioning it as a key regulator of chloroplast development.

## 1. Introduction

Chloroplasts can develop directly from the undeveloped proplastids or from etioplasts; their intermediate structures form in the absence of light [1]. During this process, thylakoids are formed and stacked into defined grana. Thylakoids are crucial structures in chloroplasts, which serve as the primary sites for the light-dependent reactions of photosynthesis. Another hallmark of chloroplast generation is chlorophyll biosynthesis. Chlorophyll is synthesized from glutamate, which is converted to 5-aminolevulinic acid (ALA) by the sequential reactions of Glu-tRNA synthetase (GluRS), glutamyl-tRNA reductase (GluTR), and glutamate 1-semialdehyde aminotransferase (GSAT) [2,3]. Among these enzymes, GluTR represents the first rate-limiting step in ALA synthesis. In Arabidopsis, GluTR enzymes are encoded by three nuclear genes, *HEMA1*, *HEMA2*, and *HEMA3*. Notably, *HEMA1* is the predominant gene, and its downregulation leads to chlorophyll deficiency, which negatively affects plant growth and development [4].

Light is a crucial environmental factor that regulates chloroplast development and chlorophyll biosynthesis in plants [5]. Light-dependent pathways involve photoreceptors, such as phytochromes and cryptochromes, which perceive different wavelengths of light and transmit signals that modulate gene expression [6]. Among the downstream regulators, the bZIP transcription factor ELONGATED HYPOCOTYL 5 (HY5) acts as a central regulator of chloroplast development by integrating signals from these photoreceptors [7]. This integration is vital for the coordinated expression of genes essential for chloroplast biogenesis and function. A recent study demonstrated that HY5 directly activates the expression of two *GOLDEN2-LIKE* (*GLK*) family transcription factors, *GLK1* and *GLK2*, which are key regulators of chloroplast biogenesis [8,9]. These GLKs function synergistically with MYB-related transcription factors to enhance the expression of several nucleus-encoded genes critical for photosynthesis, particularly those involved in chlorophyll biosynthesis and light-harvesting capabilities [10,11]. The double mutant *glk1 glk2* exhibits a distinct pale-green phenotype and a significant reduction in thylakoid formation, underscoring the critical roles of *GLK1* and *GLK2* in chlorophyll biosynthesis and chloroplast development [12]. Conversely, an overexpression of either *GLK1* or *GLK2* induces chloroplast development in non-green tissues, suggesting their potential use in biotechnological applications aimed at enhancing photosynthetic efficiency in crop plants [13,14].

Phytohormones, particularly cytokinins, play an essential role in chloroplast biogenesis and function [15]. The involvement of cytokinins in delaying chlorophyll degradation was recognized soon after their identification as plant growth regulators [16]. Over the past two decades, significant insights into the molecular pathways governing cytokinin-mediated chloroplast development have emerged. In Arabidopsis, cytokinin is perceived by three histidine kinases: ARABIDOPSIS HIS KINASE2 (AHK2), AHK3, and CRE1/AHK4, which function as cytokinin receptors. Upon cytokinin binding, these receptors transduce signals to type-B Arabidopsis response regulators (type-B ARRs) through histidine phosphotransfer proteins (AHPs) [17]. Type-B ARRs subsequently activate the transcription of downstream target genes that regulate chloroplast development [18]. Among these receptors, AHK3 plays a pivotal role in mediating the effects of cytokinins on chloroplast development and chlorophyll accumulation. Mutations in *AHK3* result in an approximately 25% reduction in chlorophyll content compared to wild-type plants, while mutations in *AHK2* have a negligible impact and *AHK4* mutations exhibit no effect on chlorophyll levels [19]. Furthermore, a significant reduction in chlorophyll content has been observed in the triple type-B ARR mutant *arr1 arr10 arr12* [18], indicating that the target genes of these type-B ARRs are essential for chloroplast development. Notably, ARR10 and ARR12 specifically bind to the promoters of *HEMA1* and *LIGHT HARVESTING COMPLEX PHOTOSYSTEM II SUBUNIT6* (*LHCB6*) [2]. *HEMA1* and *LHCB6* encode glutamyl-tRNA reductase and Glu-1-semialdehyde aminotransferase, respectively, which catalyze the initial rate-limiting steps of chlorophyll synthesis.

Chloroplast differentiation in roots is mechanistically different from that in aerial tissues; however, the regulatory network governing root chloroplast development can be equally complex, comprising multiple transcription factors. Kobayashi et al. [20] have reported that the B-GATA transcription factor *GNC-LIKE/CYTOKININ-RESPONSIVE GATA1* (*GNL*) is significantly upregulated in detached roots mediated by ARR1 and ARR12. Although *GNL* does not directly participate in chlorophyll synthesis, its overexpression promotes ectopic chloroplast development, while a loss of *GNL* function results in reduced chlorophyll accumulation. Collectively, these findings suggest that *ARR1*, *ARR10*, and *ARR12* functionally overlap and act as key regulators of cytokinin signaling during chloroplast development and chlorophyll accumulation. In Arabidopsis, there are 11 type-B ARRs that can be categorized into three subfamilies based on phylogenetic analysis. However, other than *ARR1*, *ARR10*, and *ARR12*, the roles of the remaining type-B ARRs in chloroplast development remain largely unexplored.

During embryogenesis, the formation of chloroplasts from proplastids is as critical as their development in vegetative organs. However, the regulatory mechanisms of chloroplast development and chlorophyll biosynthesis in reproductive organs remain poorly understood. In a previous study, we reported that overexpression of the seed-specific R2R3-MYB transcription factor PGA37 induces the formation of somatic embryos in Arabidopsis [21]. In this study, we further investigated the role of PGA37 in chloroplast development, emphasizing its essential function in regulating chlorophyll accumulation in seeds and chloroplast biogenesis in roots through the direct regulation of *GLK2*, *ARR13*, and *ARR21*.

## 2. Results

### 2.1. Ectopic Expression of PGA37 Induces Chloroplast Development in Roots

**Figure 1:**
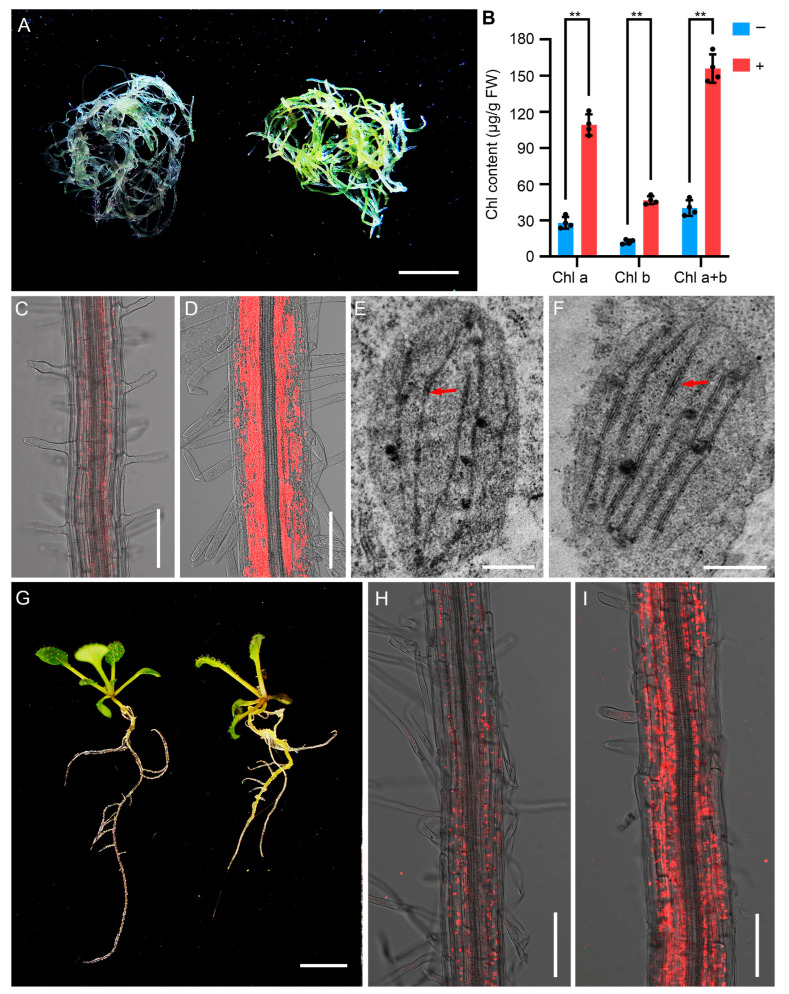
Chloroplasts in *pga37* roots. (**A**) The green root phenotype of the *pga37* mutant. Roots were detached from two-week-old *pga37* seedlings that were germinated and grown on MS medium, either in the absence (**left**) or presence (**right**) of 10 μM estradiol. (**B**) Chlorophyll content in the roots of panel (**A**). Error bars indicate the standard deviation (*n* = 4), and ** denotes a significance level of *p* < 0.01, based on Student’s *t*-test. (**C**,**D**) Chlorophyll fluorescence in the primary roots of two-week-old *pga37* seedlings germinated and grown on MS medium, either without (**C**) or with 10 μM (**D**) estradiol. (**E**,**F**) The ultrastructure of plastids in the primary root cells of *pga37* seedlings shown in panel (**C**,**D**). The structures indicated by the red arrows are grana stacks. (**G**) Two-week-old pER10-*PGA37* transgenic seedlings grown on MS medium with either 0 μM (**Left**) or 0.1 μM (**Right**) estradiol. (**H**,**I**) Chlorophyll fluorescence in the primary roots of two-week-old pER10-*PGA37* transgenic seedlings germinated and grown on MS medium with either 0 (**H**) or 0.1 μM (**I**) estradiol. Scale bars: 5 mm in (**A**,**G**); 0.1 mm in (**C**,**D**,**H**,**I**); 0.5 μm in (**E**,**F**).

In higher plant roots, chloroplast development is typically suppressed even under light conditions, resulting in roots that are generally white or yellowish (Figure 1A). However, the roots of the *plant growth activator 37* (*pga37*) mutant exhibit a distinct light-green phenotype, indicating the formation of chloroplasts in *pga37* roots (Figure 1A). The *pga37* is a gain-of-function mutant in which the ectopic expression of the seed-specific *PGA37* gene can be induced by estradiol [21]. Quantitative analysis revealed that the total chlorophyll content in roots of *pga37* seedlings grown on an inductive medium with estradiol is approximately 3.9 times higher than that of roots on non-inductive medium (Figure 1B). Further chlorophyll autofluorescence analysis revealed that chlorophyll was predominantly accumulated in the thickened stele of the *pga37* primary root (Figure 1C,D). Additionally, chlorophyll accumulation was also observed in the outer cell layers, endodermis, and cortex (Figure 1C,D). A similar distribution pattern of chlorophyll-containing cells was noted in the roots of *GLK1* or *GLK2* overexpression lines [13]. To ascertain whether chloroplasts developed in the green root of the *pga37* or not, an ultrastructural analysis of *pga37* roots was conducted using transmission electron microscopy. In the absence of estradiol, *pga37* seedlings exhibited poorly developed thylakoid membrane networks in root plastids (Figure 1E). Conversely, root plastids exposed to 10 μM estradiol displayed well-formed thylakoid membranes and grana structures (Figure 1F).

To further confirm that the green root phenotype was attributable to the gain-of-function mutation in *PGA37*, we examined the phenotype of pER10-*PGA37* transgenic seedlings. Given that the growth and development of pER10-*PGA37* transgenic plants were completely inhibited at higher concentrations of the inducer, we opted to germinate the seeds on MS medium supplemented with 0.1 μM 17-β-estradiol. The green root phenotype observed in *pga37* gain-of-function mutant was successfully replicated in the pER10-*PGA37* transgenic plants (Figure 1G). Furthermore, the chlorophyll distribution pattern in the roots of pER10-*PGA37* transgenic seedlings closely mirrored that observed in the roots of *pga37* mutants (Figure 1H,I), reinforcing the causal link between *PGA37* overexpression and the observed chlorophyll accumulation. (Figure 1H,I).

### 2.2. Light Enhances the Regulation of Chloroplast Development by PGA37

**Figure 2:**
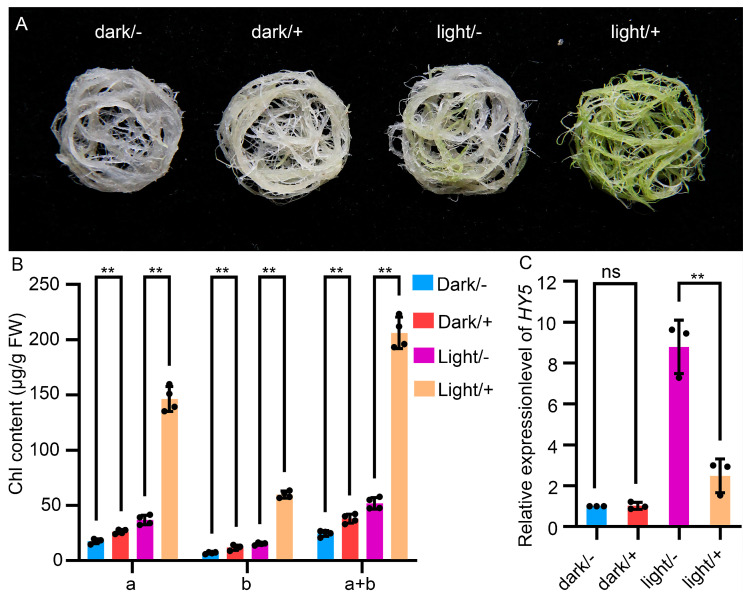
Light independent chloroplast development in *pga37* roots. (**A**) Detached roots of *pga37* seedlings cultured for 7 days either in light or in darkness, with 0 (−) or 10 (+) μM estradiol. (**B**) Chlorophyll content in *pga37* detached roots shown in Panel A. Error bars indicate the standard deviation (*n* = 4), and ** denotes a significance level of *p* < 0.01, based on Student’s *t*-test. (**C**) qRT-PCR analysis of *HY5* in detached roots of *pga37* seedlings shown in panel (**A**). Error bars indicate the standard deviation (*n* = 3), and ** denotes a significance level of *p* < 0.01; ns, not significant, Student’s *t*-test. Scale bar, 1 cm in (**A**).

Previously, Kobayashi et al. [22] reported that light signaling was required for the greening of detached roots. To investigate whether chloroplast development in the *pga37* mutant depends on light, detached *pga37* roots were cultured on MS medium containing 10 μM 17-β-estradiol under both light and dark conditions. The results demonstrated that the application of estradiol induced a greening phenotype in the *pga37* mutant roots under both conditions (Figure 2A). However, it was observed that the roots accumulated significantly more chlorophyll when estradiol was applied under light conditions compared to dark conditions (Figure 2A,B). To further investigate the influence of light on *PGA37*, we examined the effect of *PGA37* overexpression on the expression of *HY5*, a key regulatory gene in the light signaling pathway. Under dark conditions, *PGA37* overexpression had no significant impact on *HY5* expression (Figure 2C). However, under light conditions, *HY5* expression was notably inhibited by *PGA37* (Figure 2C). These findings suggest that PGA37 requires light to maximize its activities in root greening and that it also influences the expression of *HY5*.

### 2.3. Genome-Wide Identification of the Potential Targets of PGA37 Using a Glucocorticoid-Inducible System

**Figure 3:**
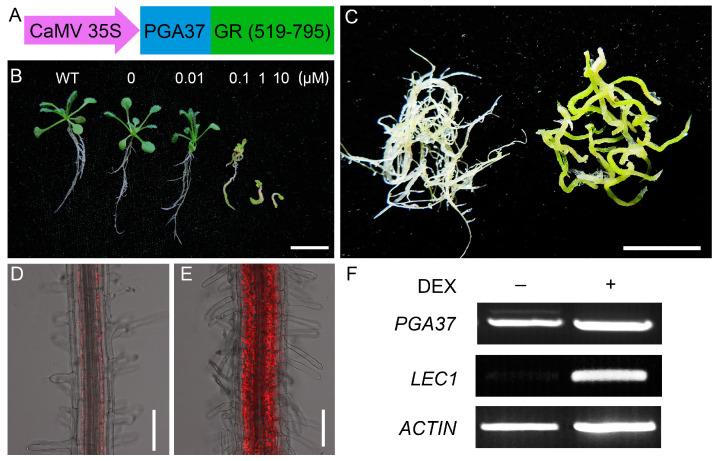
Generation and characterization of *35S*:*PGA37-GR* transgenic plants. (**A**) Diagram of the *35S*:*PGA37*-*GR* fusion construct. (**B**) Two-week old *35S*:*PGA37*-*GR* transgenic seedlings germinated and grown on MS medium containing varying concentrations of DEX, as indicated above. (**C**) Roots of two-week old *35S*:*PGA37*-*GR* fusion transgenic seedlings germinated and grown on MS medium with 0 μM DEX (**left**) or 0.1 μM (**right**) DEX. (**D**,**E**) Regiospecific accumulation of chlorophyll in the primary root of two-week-old *35S*:*PGA37*-*GR* seedlings on MS medium with (**right**) or without (**left**) 0.1 μM DEX. (**F**) Expression of *PGA37* and *LEC1* in *35S*::*PGA37-GR* transgenic seedlings germinated and grown on MS medium for 2 weeks and then treated with (+) or without (−) 10 μM DEX for 12 h. Scale bars, 1 cm in (**B**,**C**); 0.1 mm in (**D**,**E**).

To further elucidate the role of PGA37 in chloroplast development, we conducted RNA-Seq analysis to identify the direct target genes of PGA37, utilizing a glucocorticoid receptor (GR)-based inducible system *35S*::*PGA37*-*GR* (Figure 3A). In this system, the activation of PGA37 target genes was independent of protein synthesis when the *35S*::*PGA37*-*GR* transgenic seedlings were treated simultaneously with dexamethasone (DEX) and cycloheximide (CHX) [23]. Therefore, this suggests a direct relationship between the treatment and the activation of PGA37 target genes. In the absence of DEX induction, the *35S*::*PGA37*-*GR* transgenic seedlings displayed phenotypes that were indistinguishable from the wild-type. However, upon DEX induction, the phenotypes of *pga37* mutant and pER10-*PGA37* transgenic seedlings were recapitulated in *35S*::*PGA37*-*GR* transgenic plants, including root-greening, the distribution pattern of chlorophyll-containing cells, the retarded growth and development of the transgenic seedlings, and the somatic embryo formation of the root explants (Figure 3B–E and Figure S1). Notably, our previous research suggested that PGA37 acts upstream from *LEC1* to regulate the vegetative-to-embryonic transition [21]. Supporting this, we observed that *LEC1* expression was rapidly induced by DEX in the *35S*::*PGA37*-*GR* transgenic seedlings (Figure 3F). Collectively, these results demonstrate that the PGA37-GR fusion protein retains its biological activity and effectively modulates the expression of its target genes upon DEX induction, thereby confirming its role in promoting chloroplast development.

The *35S*::*PGA37*-*GR* transgenic seedlings were grown under normal conditions for 2 weeks and then treated with DEX and CHX. Subsequently, RNA-Seq was employed to analyze gene expression patterns. The RNA-Seq data revealed 97 differentially expressed genes, with 90 upregulated and 7 downregulated (Table S1). Since the secondary transcriptional regulation downstream of PGA37 was prevented by CHX, these genes are potential direct targets of PGA37. Most of the differentially expressed genes are involved in several biological processes, including porphyrin metabolism, fatty acid biosynthesis, fatty acid metabolism, and cytokinin signal transduction (Figures S2 and S3). Consistent with our previous findings that an overexpression of *PGA37* leads to lipid accumulation [21], we observed a significant upregulation of *AtDES3*, a key gene involved in oleic acid synthesis, in the *35S*::*PGA37*-*GR* transgenic seedlings following DEX treatment (Table S1). Further analysis revealed that 88 of the 90 upregulated genes and all downregulated genes contained at least one MYB binding sequence in their 1.5 kb upstream regions (Figure S4). Among the two exceptions, *AT5G57480* encodes a P-loop containing nucleoside triphosphate hydrolases superfamily protein, while *AT2G13910* is a pseudogene and its 1.5 kb upstream region could not be obtained.

### 2.4. PGA37 Induces Chloroplast Development by Directly Regulating the Expression of GLK2 but Not GLK1

**Figure 4:**
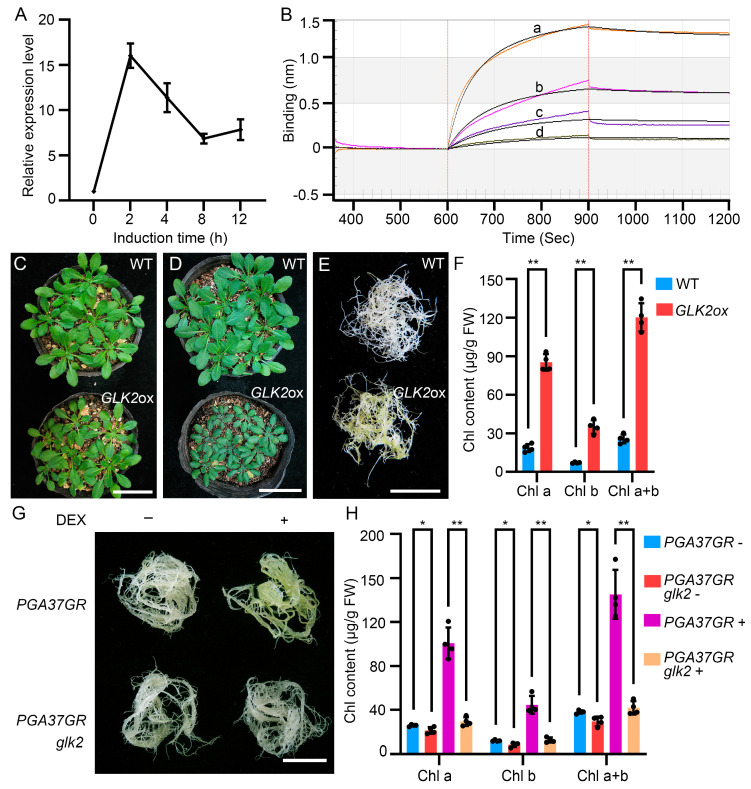
PGA37 induces chloroplast development by directly binding to the *GLK2* promoter. (**A**) Expression level of *GLK2* in the *35S*::*PGA37-GR* transgenic seedlings treated with 10 μM DEX for various times, as indicated. (**B**) Binding curve of PGA37 with *GLK2*. The lowercase letters a, b, c, and d marked on the curve represent the PGA37 protein concentrations, which are 800 nM, 400 nM, 200 nM, and 100 nM, respectively. (**C**,**D**) Four-week-old seedlings of wildtype (WT) and *GLK2ox* grown under normal (~120 μmol m^−2^ s^−1^) or high light (~800 μmol m^−2^ s^−1^) conditions. (**E**) The roots of two-week-old WT and *GLK2ox* seedlings germinated and grown on MS medium. Note that the *GLK2ox* transgenic plants showed a *pga37-*like green root phenotype. (**F**) Chlorophyll content in roots shown in panel (**F**). Error bars indicate standard deviation (*n* = 4), ** indicates *p* < 0.01, Student’s *t*-test. (**G**) Roots of two-week-old *35S*::*PGA37-GR* and *35S*::*PGA37-GR glk2* seedlings germinated on MS medium with (+) or without (−) DEX. (**H**) Chlorophyll content in the roots shown in panel (**G**). Error bars represent the standard deviation (*n* = 4). A single asterisk (*) denotes 0.01 < *p* < 0.05, while a double asterisk (**) indicates *p* < 0.01. Scale bars: 5 cm in (**C**,**D**), 1 cm in (**E**,**F**).

Among the 97 potential *PGA37* target genes, we found that the *GLK2* gene showed a significant upregulation in seedlings of *35S*::*PGA37*-*GR* upon DEX induction (Figure 4A and Table S1). Therefore, the rapid activation of *GLK2* by PGA37 suggests a direct regulatory mechanism, likely involving the interaction of PGA37 with the *GLK2* promoter. To further substantiate the direct interaction between PGA37 and the *GLK2* promoter, we conducted biolayer interferometry (BLI) assays. As depicted in Figure 4B, PGA37 exhibited a strong affinity for the *GLK2* promoter region, with a dissociation constant (Kd) of 2.32 × 10 ^−7^ M, thereby confirming the direct binding of PGA37 to the *GLK2* promoter.

To further investigate the role of *PAG37* in chloroplast development, we characterized the phenotype of *GLK2* overexpression lines (*GLK2ox*) and examined the effect of its mutation on chlorophyll accumulation in the roots of *35S*::*PGA37*-*GR* seedlings. Under normal light conditions, the aerial parts of *GLK2ox* showed no significant differences compared to the wild type (Figure 4C). However, under high light conditions, *GLK2ox* accumulates anthocyanins in the aerial parts and exhibited stunted growth and development (Figure 4D). Notably, the roots of *GLK2* overexpression seedlings showed a significant increase in chlorophyll accumulation, resulting in a root-greening phenotype similar to that observed in *pga37* mutants and pER10-*PGA37* transgenic seedlings (Figure 4E,F). Conversely, the root-greening phenotype induced by PGA37 overexpression was suppressed by the mutation of the *GLK2* gene (Figure 4G,H). These findings clearly demonstrate that PGA37 induces chloroplast development by promoting the expression of *GLK2*.

In Arabidopsis, two *GLK* genes, *GLK1* and *GLK2*, function redundantly to regulate chloroplast development [12,24]. Additionally, an overexpression of *GLK1* has been reported to induce a green root phenotype similar to that observed with *GLK2* overexpression [13]. Given this, it is possible that *GLK1* might also play a role in PGA37-mediated chloroplast development. However, our analysis revealed that *GLK1* expression was not induced in *PGA37* overexpression seedlings, suggesting that *GLK1* is dispensable for the root-greening phenotype observed in *35S*::*PGA37*-*GR* transgenic lines (Table S1 and Figure S5a). Moreover, BLI assay indicates that PGA37 protein could not bind to the *GLK1* promoter (Figure S5b). These findings indicate a specific regulatory role for *GLK2* in the *PGA37*-mediated pathway, underscoring the distinct contribution of *GLK2* to chloroplast development under the influence of PGA37.

### 2.5. PGA37 Promotes Root-Greening Partially Through Cytokinin Signaling Activated by ARR13 and ARR21

**Figure 5:**
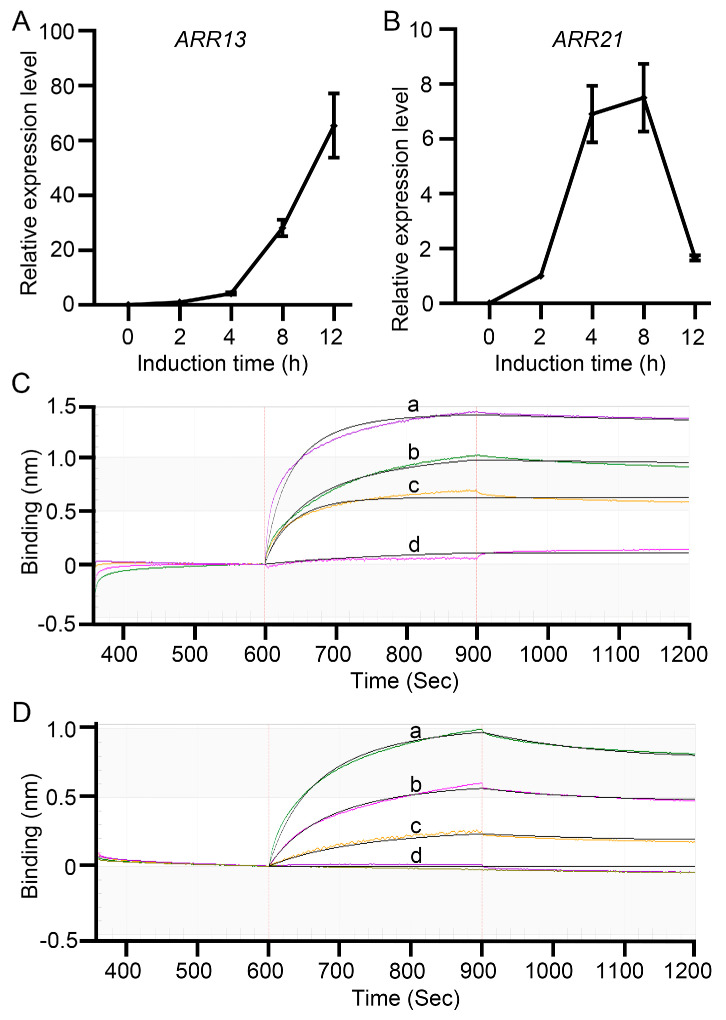
PGA37 directly binds to the promoter region of *ARR13* and *ARR21.* (**A**,**B**) Expression of *ARR13* (**A**) and *ARR21* (**B**) in *35S*::*PGA37-GR* transgenic seedlings treated with 10 μM DEX for various times. (**C**,**D**) Binding curves of PGA37 with *ARR13* (**C**) and *ARR21* (**D**). The lowercase letters a, b, c, and d marked on the curve represent the PGA37 protein concentrations, which are 800 nM, 400 nM, 200 nM, and 100 nM, respectively.

We observed a significantly lower chlorophyll content in the roots of *GLK2* overexpression (*GLK2ox*) plants compared to *pga37* mutants (Figure 1B and Figure 4F). Moreover, our previous finding indicated that *PGA37* can promote the vegetative-to-embryonic transition independently of cytokinin [21]. These findings led us to hypothesize that the root-greening phenotype in *pga37* mutants might be at least partially mediated by cytokinin signaling activated by *PGA37*. Supporting this hypothesis, we found that two type-B *ARR* genes, *ARR13* and *ARR21*, were significantly induced by DEX in *35S*::*PGA37*-*GR* transgenic lines (Table S1, Figure 5A,B). Further BLI assays confirm the direct interaction between PGA37 transcription factor and the fragments containing the upstream regulatory sequences near the translational start sites of both *ARR13* and *ARR21* (Figure 5C,D).

**Figure 6:**
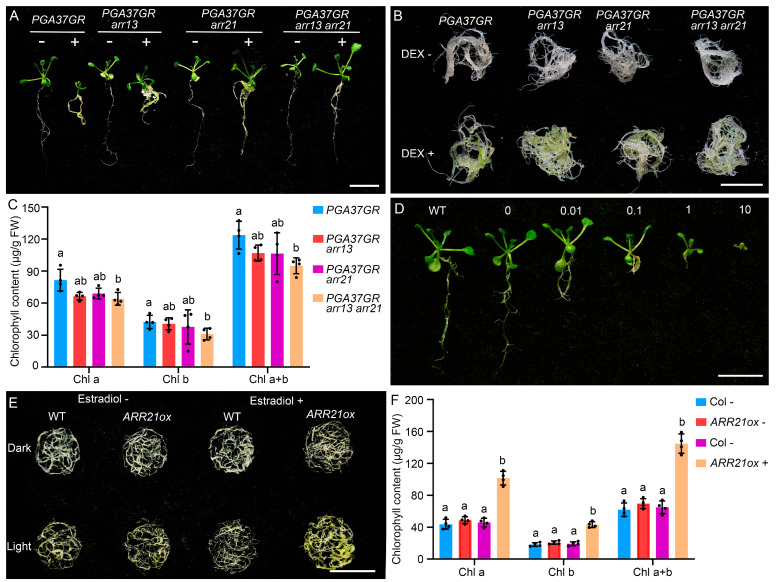
Functional characterization of *ARR13* and *ARR21* genes in regulating plant growth and chlorophyll biosynthesis. (**A**) Two-week-old seedlings of *35S*::*PGA37-GR*, *35S*::*PGA37-GR arr13*, *35S*::*PGA37-GR arr21*, *35S*::*PGA37-GR arr13 arr21* germinated and grown on MS medium with 0.2 μM or without DEX. (**B**) Roots of seedlings shown in panel (**A**). (**C**) Chlorophyll content in intact roots of seedlings shown in panel (**A**). (**D**) Two-week-old seedling of wild type (Col) and pER10-*ARR21* germinated and grown on MS medium supplemented with 0, 0.01, 0.1, 1 or 10 μM estradiol. (**E**) Roots of two-week-old seedlings, initially germinated and grown on MS medium, were transferred to MS medium containing either 0 (−) or 10 μM (+) estradiol. They were then cultured for one additional week under either dark or light conditions. (**F**) The chlorophyll content in roots cultured under light conditions shown in panel (**E**). Error bars in panels (**C**,**E**) indicate the standard deviation (*n* = 4). The different lowercase letters above columns indicate statistically significant differences within the same group (Chl a, Chl b, or Chl a + b), while identical lowercase letters signify no significant difference, *p* < 0.05, Student’s *t*-test. Scale bar: 1 cm.

To elucidate the dependency of the PGA37-mediated root-greening phenotype on *ARR13* and/or *ARR21*, we generated *35S*::*PGA37*-*GR arr13*, *35S*::*PGA37*-*GR arr21*, and *35S*::*PGA37*-*GR arr13 arr21* lines by crossing the *arr13 arr21* double mutant with *35S*::*PGA37*-*GR* transgenic line. Surprisingly, a single mutation in either *ARR13* or *ARR21* partially suppressed the growth-retardant phenotype caused by PGA37 overexpression (Figure 6A). In contrast, a double mutation in both *ARR13* and *ARR21* nearly completely suppressed the growth-retardant phenotype. This suggests that the growth-retardant phenotype observed in PGA37 overexpression lines is due to the ectopic expression of *ARR13* and *ARR21*. Interestingly, despite the fact that the single mutation of *ARR13* or *ARR21* did not affect root chloroplast development in the *PGA37* overexpression lines, the chlorophyll content in the roots of *35S*:*PGA37-GR arr13 arr21* triple mutants was significantly decreased (Figure 6B,C). To gain deeper insight into the role of *ARR21* in plant growth and chlorophyll biosynthesis, we analyzed the phenotype of pER8-*ARR21* estrogen-inducible overexpression lines. As anticipated, inducible overexpression of *ARR21* led to a marked growth and developmental retardation, closely resembling the phenotype observed with cytokinin application (Figure 6D). Under dark conditions, there was no significant difference in chlorophyll content between the roots of pER8-*ARR21* transgenic lines treated with 10 μM estradiol and those left untreated (Figure 6E,F). However, under light conditions, the chlorophyll content was significantly higher in estradiol-treated roots compared to untreated roots. Taken together, our results demonstrate that PGA37 activates cytokinin signaling by directly regulating the expression of *ARR13* and *ARR21*, ultimately contributing to the occurrence of ectopic chloroplast development in Arabidopsis.

## 3. Discussion

**Figure 7:**
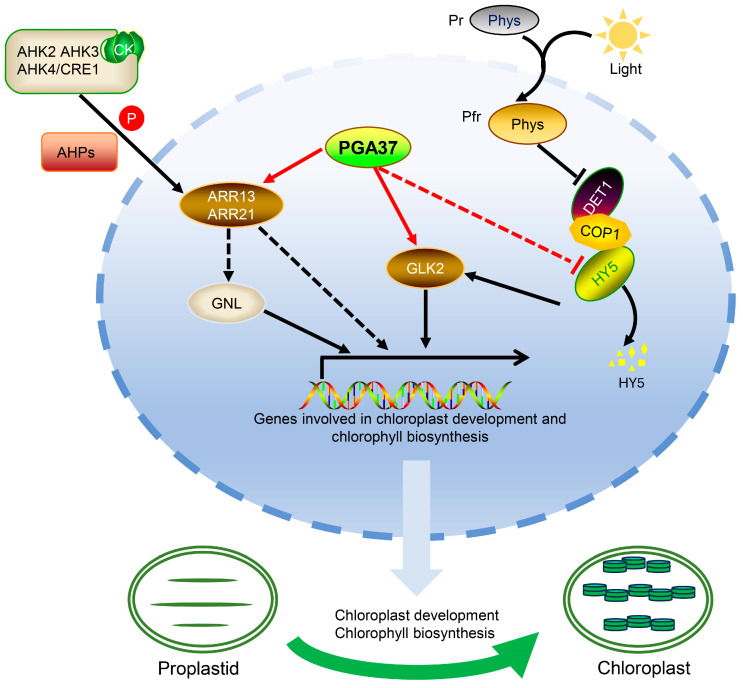
A working model illustrates the roles of *PGA37*, *GLK2*, *ARR13*, and *ARR21* in regulating chloroplast development. The R2R3-MYB transcription factor PGA37 inhibits HY5, while it activates GLK2 and cytokinin signaling mediated by ARR13 and ARR21. This coordinated action integrates with light signaling from light receptors such as phytochromes (Phys) and is mediated through the interaction of HY5, COP1, and DE-ETIOLATED 1 (DET1). ARR13 and ARR21 transmit cytokinin signals from phosphorylated AHPs and are subject to regulation through GNL or an unelucidated pathway. Arrows and T-bars indicate positive and negative transcriptional regulation, respectively. The dashed lines represent unelucidated regulation or indirect effects through unknown intermediate factors. Abbreviations: CK stands for cytokinin; Pr represents the phytochrome red-absorbing form, which is the inactive state of phytochrome. This form converts to the active Pfr (phytochrome far-red-absorbing form) upon absorbing red light and subsequently enters the nucleus. The circled P denotes phosphorylation.

Chloroplasts are essential for plant growth and development, as they enable photosynthesis and significantly impact energy production and biomass accumulation, which ultimately influence crop yield and productivity. Therefore, the targeted engineering of this process could significantly contribute to crop improvement and help to sustainably meet global food and bioenergy demands [25]. In this study, we elucidated that an overexpression of *PGA37*, a seed-specific gene, leads to the ectopic induction of chloroplasts in roots by integrating cross-family transcription factors, including those from families of MYB (PGA37/MYB118), GLK (GLK2), bZIP (HY5), and type-B ARR (ARR13 and ARR21) (Figure 7). Since chloroplasts are typically absent in roots, our findings offer potential for enhancing photosynthesis in conditions where roots are exposed to light, such as in hydroponic or soilless cultivation systems. Furthermore, the homologs of *PGA37* may play a more crucial role in epiphytic plants, such as many species within the *Orchidaceae* family, whose root systems are capable of photosynthesis [26]. This approach could lead to increased yield and productivity under these specific agricultural practices.

In higher plants, the development of chloroplasts in roots is generally suppressed, even when exposed to light [13], making the light-independent chloroplast biogenesis in *pga37* roots particularly remarkable. Previous studies, such as those by Kobayashi et al. [22], have shown that light signaling is crucial for chloroplast development in detached roots. However, our results clearly show that *PGA37* could induce chloroplast formation in *pga37* roots under both light and dark conditions, with a significant increase in chlorophyll content under light conditions (Figure 2A,B). This observation suggests a synergistic interaction between *PGA37* and light that enhances chloroplast development, although light is not strictly necessary for the process. The elevated accumulation of chlorophyll observed in the roots of *PGA37*-overexpressing plants under dark conditions may be due to the activation of *GLK2*. However, other pathways activated by *PGA37*, such as the cytokinin signaling pathway, could also contribute to chloroplast induction under dark conditions. Interestingly, we found that the expression of *HY5* was significantly repressed by *PGA37* under light conditions (Figure 2C). It has been reported that cytokinin can increase HY5 protein accumulation by reducing its CONSTITUTIVE PHOTOMORPHOGENESIS 1 (COP1)-dependent degradation [27]. We hypothesize that the activation of the cytokinin signaling pathway by *ARR13* and *ARR21* might lead to an increase in the HY5 protein levels, resulting in a negative feedback regulation of *HY5* gene expression. However, we cannot rule out the possibility that PGA37 may interact with HY5, a light-induced protein, which could also, through negative feedback, regulate the expression of *HY5*. In contrast to *PGA37′*s role in promoting chloroplast development in roots, we observed that the *35S*::*PGA37GR* and pER10-*PGA37* transgenic seedlings displayed yellowish leaves when cultivated on a medium with a relative high concentration of inducer (Figure 3B, see also reference [21] (Wang et al., 2009). We hypothesize that this reduction in leaf chlorophyll is related to the expression levels of *PGA37*. At lower expression levels, *PGA37* promotes chloroplast development. However, at excessively high expression levels, it may activate the synthesis of other products through its target genes, which, at elevated levels, could become toxic and result in chlorophyll degradation in leaves. Furthermore, the basal levels of these potentially toxic products are inherently lower in roots compared to leaves, causing a reduced-chlorophyll phenotype to appear in leaves first, where these products are naturally more abundant. In addition, the excessive production of certain intermediates or chlorophyll itself may trigger the feedback inhibition of the chlorophyll biosynthetic pathway, leading to the pale-yellow phenotype. Further experimental evidence will be required to validate this hypothesis.

The genome-wide identification of potential PGA37 target genes using a glucocorticoid-inducible system has provided valuable insights into the downstream regulatory network controlled by PGA37. RNA-Seq analysis identified 97 differentially expressed genes, with most being upregulated, indicating that PGA37 predominantly acts as a transcriptional activator (Table S1). Among these differentially expressed genes, *GLK2* was identified as a direct target, showing rapid upregulation upon the induction of PGA37 expression (Figure 4). The role of *GLK2* in chloroplast development is further supported by the phenotypic analysis of *GLK2* overexpression lines, which exhibited a root-greening phenotype similar to that of *pga37* mutants (Figure 4E,F). The suppression of this phenotype by *GLK2* mutation in PGA37 overexpression lines underscores *GLK2′*s important role in this pathway (Figure 4G,H). The lack of *GLK1* induction and the inability of PGA37 binding to the *GLK1* promoter highlight the specific and non-redundant function of *GLK2* in *PGA37*-mediated chloroplast development, suggesting that *GLK1* is dispensable in this context (Figure S5). Moreover, *GLK1* is scarcely expressed in Arabidopsis roots, while *GLK2*, but not *GLK1*, was upregulated in roots treated with cytokinin [22]. These findings consistently support the crucial role of *GLK2* in chloroplast development mediated by PGA37. In contrast, recent findings by Frangedakis et al. [11] revealed that two RR-MYBs, AtMYBS1 and AtMYBS2, can bind to the promoter of *GLK1* but not *GLK2*. This suggests a distinct regulatory mechanism involving RR-MYBs and GLK1 that may complement or diverge from the pathways mediated by PGA37 and GLK2. These insights broaden our understanding of the complex regulatory networks governing chloroplast development.

In Arabidopsis, there are 11 type-B ARRs categorized into three distinct subfamilies [28]. Previous research by Cortleven et al. [2] has identified three type-B ARRs from subfamily I—*ARR1*, *ARR10*, and *ARR12*—as critical players in chloroplast development. Notably, *ARR1* and *ARR12* have been shown to be essential for chloroplast development specifically in detached roots [20]. In our study, we discovered that ARR13 and ARR21, both from subfamily II, are direct targets of PGA37 (Figure 5). Furthermore, the significant reduction in chlorophyll content in the roots of *35S*::*PGA37*-*GR arr13 arr21* triple mutants provides compelling evidence that cytokinin signaling, mediated by *ARR13* and *ARR21*, plays a role in the ectopic development of chloroplasts in roots (Figure 6). This finding underscores the complex regulatory mechanisms orchestrated by cytokinin signaling in chloroplast development.

It is noteworthy that *ARR13* and *ARR21*, like *PGA37*, are primarily expressed in reproductive organs [21,29,30,31]. Further investigation is needed to determine whether *ARR13* and *ARR21* contribute to chloroplast development in detached roots independently of PGA37. In addition to the root-greening phenotype, *PGA37* overexpression significantly hinders seedling growth and promotes somatic embryogenesis independently of cytokinin [21]. These phenotypes may also result from the activation of the cytokinin signaling pathway by PGA37 through the upregulation of *ARR13* and *ARR21*, as the double mutation of *ARR13* and *ARR21* can nearly completely suppress the growth retardation phenotype caused by *PGA37* overexpression (Figure 6A).

The interplay of the transcriptional regulators characterized in this study reinforces the importance of cross-family transcription factor regulatory networks in gene regulation. The PGA37-regulated module includes transcription factors from different families, enabling the coordination of complex gene expression programs. Such cross-family modules allow for the integration of multiple signaling pathways, enabling cells to respond to a wide range of internal and external stimuli (e.g., hormones and light). The interaction between transcription factors from different families provides combinatorial control over gene expression. These networks often exhibit redundancy, where different transcription factors can compensate for each other’s function. While some transcription factors have broad regulatory roles, others are more specialized. During development, cross-family networks are essential for cellular differentiation and tissue-specific gene expression. Cross-family transcription factor regulatory networks are essential for the dynamic, precise, and context-dependent regulation of gene expression, enabling organisms to adapt to their environment, develop properly, and maintain homeostasis. The PGA37-mediated regulatory module integrates the phytochrome and cytokinin signals to fine-tune the expression of genes involved in chloroplast development and chlorophyll biosynthesis in various tissues (Figure 7).

## 4. Materials and Methods

### 4.1. Plant Materials and Growth Conditions

The origins of *pga37*, *myb118* (Salk_118812), *myb115* (Salk_044168), and *myb115 myb118* mutants, as well as the pER10-*PGA37* transgenic line, have been previously described [21]. The *arr13* (SALK_042719c) and *arr21* (SALK_005772c) mutants, both in the Col-0 background, were generously provided by Professor Chen Shouyi from the Institute of Genetics and Developmental Biology. The *glk2* (Salk_17006C) mutant and the *GLK2* overexpression line (*GLK2ox*, CS9906) were acquired from the Arabidopsis Biological Resource Center (ABRC, Ohio State University, Columbus, OH). The *35S*::*PGA37-GR glk2* was generated by crossing the *35S*::*PGA37-GR* overexpression line and the *glk2* mutants. To create the *arr13 arr21* double mutant, *arr13* and *arr21* were crossed, and this double mutant was subsequently crossed with *35S*::*PGA37*-*GR* to produce *35S*::*PGA37-GR arr13*, *35S*::*PGA37-GR arr21*, and *35S*::*PGA37-GR arr13 arr21* mutants. PCR-based genotyping was employed to identify homozygous lines, with the primer sequences listed in Table S2.

For the light-independent analysis of chloroplast induction, seven-day-old seedlings grown on solid MS medium were transferred to liquid MS medium and cultured for an additional two days. The roots were then excised and cultured for seven days on MS medium, either supplemented with 10 μM 17 β-estradiol (Sigma-Aldrich, St. Louis,MO, USA, Cat# E8875) or without it, under both normal light and dark conditions.

Unless otherwise specified, Arabidopsis plants were grown at 22 °C under a 16 h photoperiod in soil or on agar plates containing half-strength Murashige and Skoog basal medium with vitamins (PhytoTechnology Laboratories^TM^, St Lenexa, KS, USA, Cat# M519), supplemented with 2% sucrose and 0.8% agar.

### 4.2. Plasmid Constructs and in Planta Transformation of Arabidopsis

To make the *35S*:*PGA37-GR* fusion construct, a PCR fragment encoding the glucocorticoid receptor domain (amino acids 519 to 795) [23,32] was firstly amplified using primers GRF and GRB and introduced into the *Sma* I-*Spe* I sites of the HA-pBA plasmid. The resulting construct was designated HA-GR-pBA. Then, the *PGA37* coding sequence without stop codon was inserted into the HA-GR-pBA vector using the *Asc* I and *Spe* I sites to generate the *35S*:*PGA37-GR* construct. The primers used for plasmid construction are listed in Table S2. All constructs were thoroughly verified through extensive restriction digests and DNA sequencing analysis.

Arabidopsis transformation was performed using the floral-dip method [33] with the Agrobacterium GV3101 strain. T1 transgenic plants were selected on half-strength MS solid medium with kanamycin or Basta. Homozygous transgenic plants were obtained through self-crossing and subsequently used for analysis.

### 4.3. Chlorophyll Autofluorescence Detection and Ultrastructure Analysis of Root Plastids

To detect chlorophyll autofluorescence, the primary roots of two-week-old seedlings were examined approximately 1.5 cm from the root–hypocotyl junction using a laser confocal microscope (Leica, TCS SP8, Wetzlar, Germany). Chlorophyll autofluorescence was detected between 660 and 700 nm under 488 nm laser excitation and was merged with differential interference contrast images. For transmission electron microscopy analysis, the primary roots of two-week-old seedlings were cut approximately 1.5 cm from the root–hypocotyl junction and quickly prefixed overnight in 2.5% glutaraldehyde at 4 °C. The samples were then washed and postfixed in 1% OsO_4_ for about 4 h. After dehydration through a graded ethanol series (30%, 50%, 70%, 90%, and 100%, three times each, *v*/*v*), the samples were embedded in Epon812 resin. Ultra-thin sections, sliced to a thickness of 70 nm, were subsequently stained with 2% (*w*/*v*) uranyl acetate and lead citrate. Finally, the sections were photographed using a JEM-1400 electron microscope (JEOL Ltd., Tokyo, Japan).

### 4.4. Chlorophyll Determination

To determine root chlorophyll content, 50 mg root samples were ground in 500 μL of 80% (*v*/*v*) acetone using a tissue lyser (Scientz-48, Scientz, Ningbo, China). The mixture was then centrifuged for 5 min at 10,000 g. The resulting supernatant was transferred to a new microtube, and the remaining precipitate was re-extracted with an additional 500 μL of 80% (*v*/*v*) acetone. The absorbance of the combined supernatants was measured at 646 and 663 nm using a BioSpectrometer^®^ kinetic spectrophotometer (Eppendorf). The chlorophyll (a and b) concentrations were calculated as described by Wellburn [34].

For the determination of seed chlorophyll content, fifty seeds at the torpedo stage were homogenized in 100 μL of dimethylsulfoxide (DMSO). The resulting extracts were then centrifuged for 5 min at 10,000× *g*. The supernatant was subsequently used to assess chlorophyll content. To identify the developmental stage, seeds from the middle of the silique were examined using a BX51 microscope (Olympus, Tokyo, Japan), while the other seeds within the siliques were utilized for chlorophyll measurement.

Chlorophyll assessments were conducted in three independent experiments for embryos and four independent experiments for roots.

### 4.5. RNA Isolation, cDNA Synthesis, and Quantitative Real-Time PCR

Total RNA was extracted using the RNAprep pure Plant Kit with DNase I (Tiangen Biotech (Beijing), China, Cat# DP432). First-strand cDNA synthesis was performed using the PrimeScript™ II 1st Strand cDNA Synthesis Kit (Takara Biotechnology, Dalian, China). The resulting cDNA was diluted 10- to 100-fold, and quantitative transcript analysis was conducted on a Bio-Rad CFX96 system using SYBR^®^ Fast qPCR Mix (Takara Biotechnology, Dalian, China, Cat# RR430). All procedures were carried out according to the manufacturer’s instructions.

### 4.6. Dexamethasone Treatment, Library Construction, RNA-Seq and Differential Expressed Gene Analysis

Two-week-old *35S*:*PGA37-GR* seedlings, geminated and grown on solid MS medium, were transferred to liquid MS medium containing 10 μM DEX (Sigma, Cat# D4902) and 100 μM CHX (MedChemExpress, Cat# HY-12320). Seedlings treated with 100 μM CHX were used as the control. After 4 h of treatment, the seedlings were collected and then total RNA was extracted, as described above. Library construction, RNA-Seq, and differentially expressed gene analysis were performed as described previously [35].

### 4.7. Protein Expression of PGA37 and Biolayer Interferometry Assay

To express the PGA37 protein, the coding sequence (CDS) of PGA37 was cloned into the *Nde*I and *Eco*RI restriction sites of the pET-28a (+) vector, allowing for the expression of PGA37 with an N-terminal histidine tag for affinity purification and immunoblotting. The constructed pET28-PGA37 plasmid was then transformed into BL21 (DE3) competent cells. The transformed cells were cultured induced with 1 mM isopropyl-β-d-1-thiogalactopyranoside (IPTG) at 37 °C. After incubation, the cells were harvested, and the expressed protein was purified using standard purification techniques.

The purified PGA37 protein was then subjected to a biolayer interferometry (BLI) assay to evaluate its binding interactions with various oligonucleotides using a biolayer interferometry system (BLItz, FortèBIO Inc., Fremont, CA, USA). 5′-Biotinylated DNA oligonucleotides were synthesized by Sangon Biotech Co., Ltd. (Shanghai, China), with the sequences detailed in Table S2. The biotinylated oligonucleotides were immobilized onto streptavidin biosensor tips that had been pre-wetted for 10 min in kinetic buffer containing PBS, 0.1% BSA, 0.02% Tween-20, and 0.05% sodium azide. The assay steps were initial baseline (60 s), loading (300 s), baseline stabilization (240 s), ligand–analyte association (300 s), and ligand–analyte dissociation (300 s).

## 5. Conclusions

In summary, our findings highlight the multifaceted role of *PGA37* in regulating chloroplast development in Arabidopsis. We demonstrated that ectopic expression of PGA37 induces chloroplast formation in roots through *GLK2* and cytokinin signaling mediated by *ARR13* and *ARR21*, providing new insights into the intricate regulatory networks that govern plant developmental processes. Future studies should focus on delineating the specific roles of *ARR13* and *ARR21* in chloroplast development and investigating potential downstream targets that mediate the effects of *PGA37* beyond its currently known functions. Additionally, exploring the potential of *PGA37* for manipulating this pathway in agricultural crops could offer valuable strategies for enhancing growth and yield under varying environmental conditions.

## Data Availability

The raw RNA-seq data have been deposited in the Beijing Institute of Genomics Data Center (https://bigd.big.ac.cn/) under the BioProject accession PRJCA029442.

## Supplementary Materials

The following supporting information can be downloaded https://www.mdpi.com/article/10.3390/plants14091270/s1. Figure S1: Somatic embryos generated from *35S*::*PGA37*-*GR* root explants. Figure S2: Enriched GO terms for biological processes of the putative PGA37 target genes. Figure S3: Enriched KEGG pathways of the putative PGA37 target genes. Figure S4: Putative MYB Binding sites in the 1.5-kb promoter region. Figure S5: PGA37 was unable to activate the expression of *GLK1*. Table S1: Putative PGA37 targets identified by RNA-Seq. Table S2: Primers used in this study.
