# Supplementary material for: PGA37 Overexpression Promotes Chloroplast Development in Arabidopsis Roots Through Direct Transcriptional Activation of GLK2, ARR13, and ARR21
Source: Plants (Basel). 2025 Apr 22;14(9):1270. doi: 10.3390/plants14091270 (PMC12073186; doi:10.3390/plants14091270)
Supplement: Supplementary file 1 [file plants-14-01270-s001.zip › Supplementary Information.pdf]

Supplementary Files for

PGA37 Overexpression Promotes Chloroplast Development in Arabidopsis Roots through Direct Transcriptional Activation of *GLK2*, *ARR13*, and *ARR21*

Yunfeng Wei[1,a], Huiping Yang[1], Yujing Wang[1], Huimin Shen[2], Shuwei Zhang[2], Zhirong Yang[3,4]*, Ling Yuan[3]*, Xingchun Wang[1]*

1. Houji Laboratory in Shanxi Province, College of Life Sciences, Shanxi Agricultural University, Taigu 030801, China
2. College of Agriculture, Shanxi Agricultural University, Taiyuan 030031, China
3. Department of Plant and Soil Sciences, Kentucky Tobacco Research and Development Center, University of Kentucky, Lexington, KY 40546, USA
4. Department of Basic Sciences, Shanxi Agricultural University, Taigu 030801, China

***Corresponding authors:** Zhirong Yang: zryangsx@163.com; Ling Yuan: lyuan3@uky.edu；Xingchun Wang: wxingchun@sxau.edu.cn

This file includes:

Supplementary Figures S1–S5

Supplementary Figure Legends

Caption for Table S1 and S2

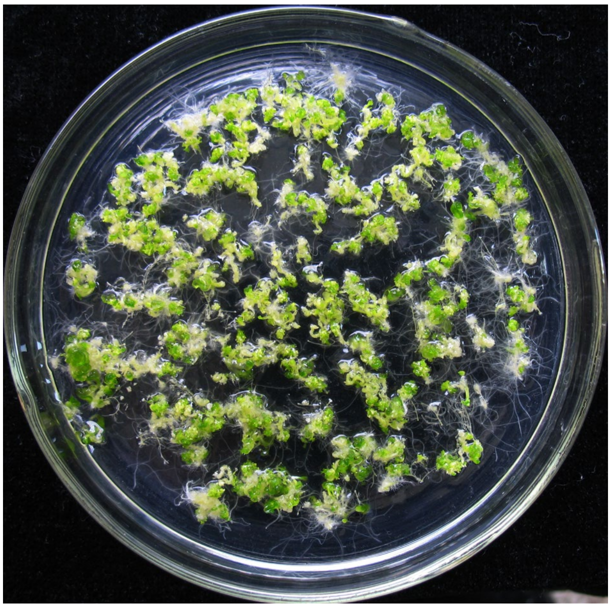

Figure S1. Somatic embryos generated from *35S*::*PGA37-GR* root explants. Roots of *35S*::*PGA37-GR* transgenic seedlings were excised and cultured on screening medium (containing 1×MS salts, 1% sucrose, 0.5 g/L MES, 0.15 mg/L IAA, 5 µm 17-β-estradiol, auxin, 17-β-estradiol) for 21 days. Bar: 1 cm.

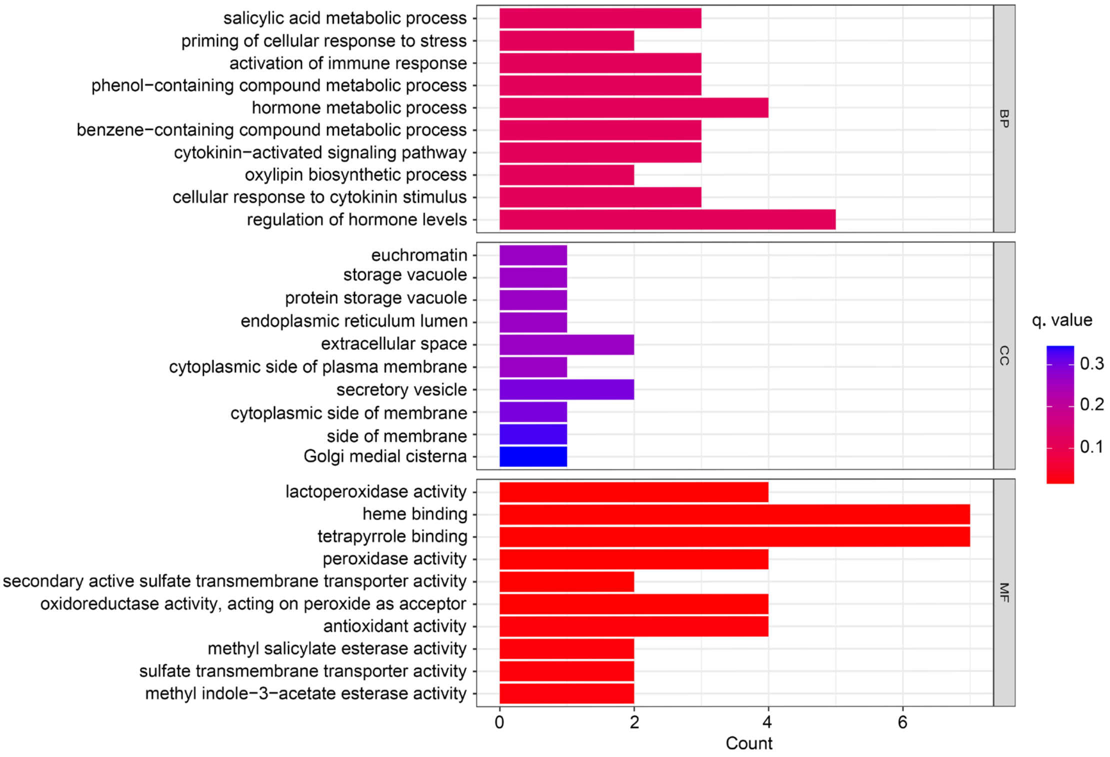

Figure S2 Enriched GO terms for biological processes of the putative PGA37 target genes.

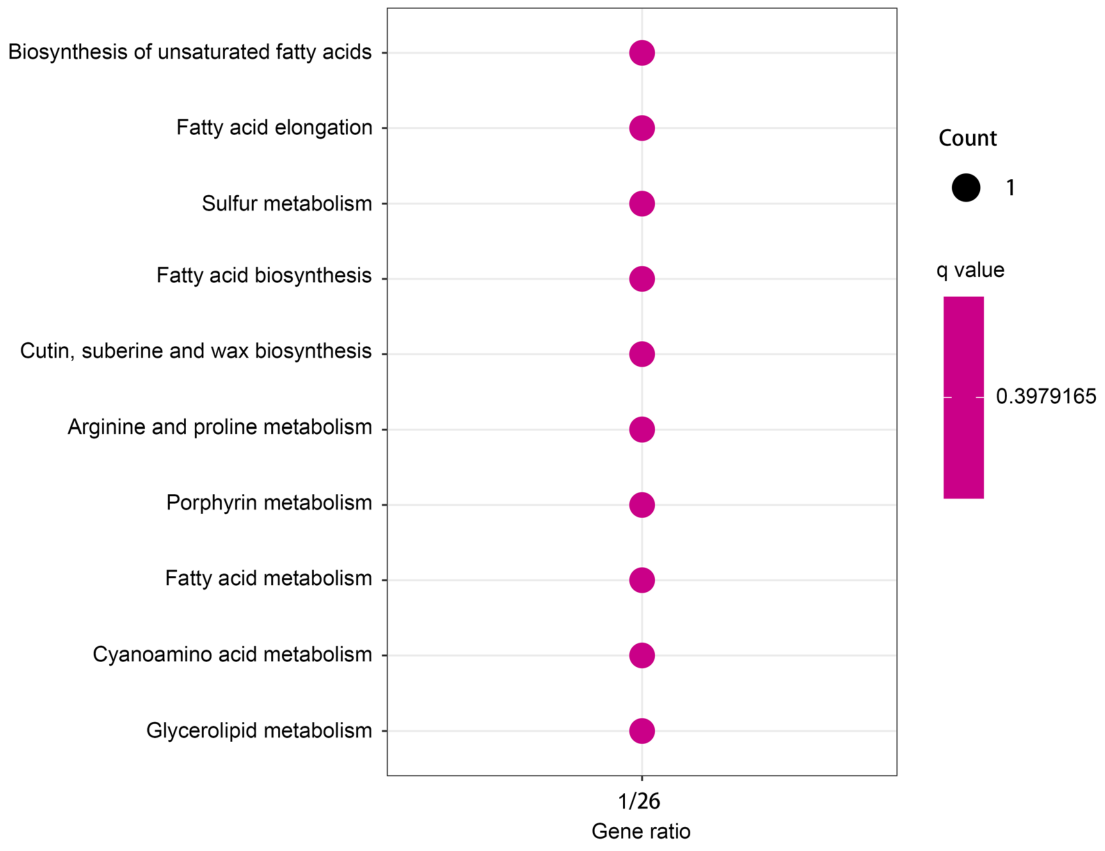

Figure S3. Enriched KEGG pathways of the putative PGA37 target genes

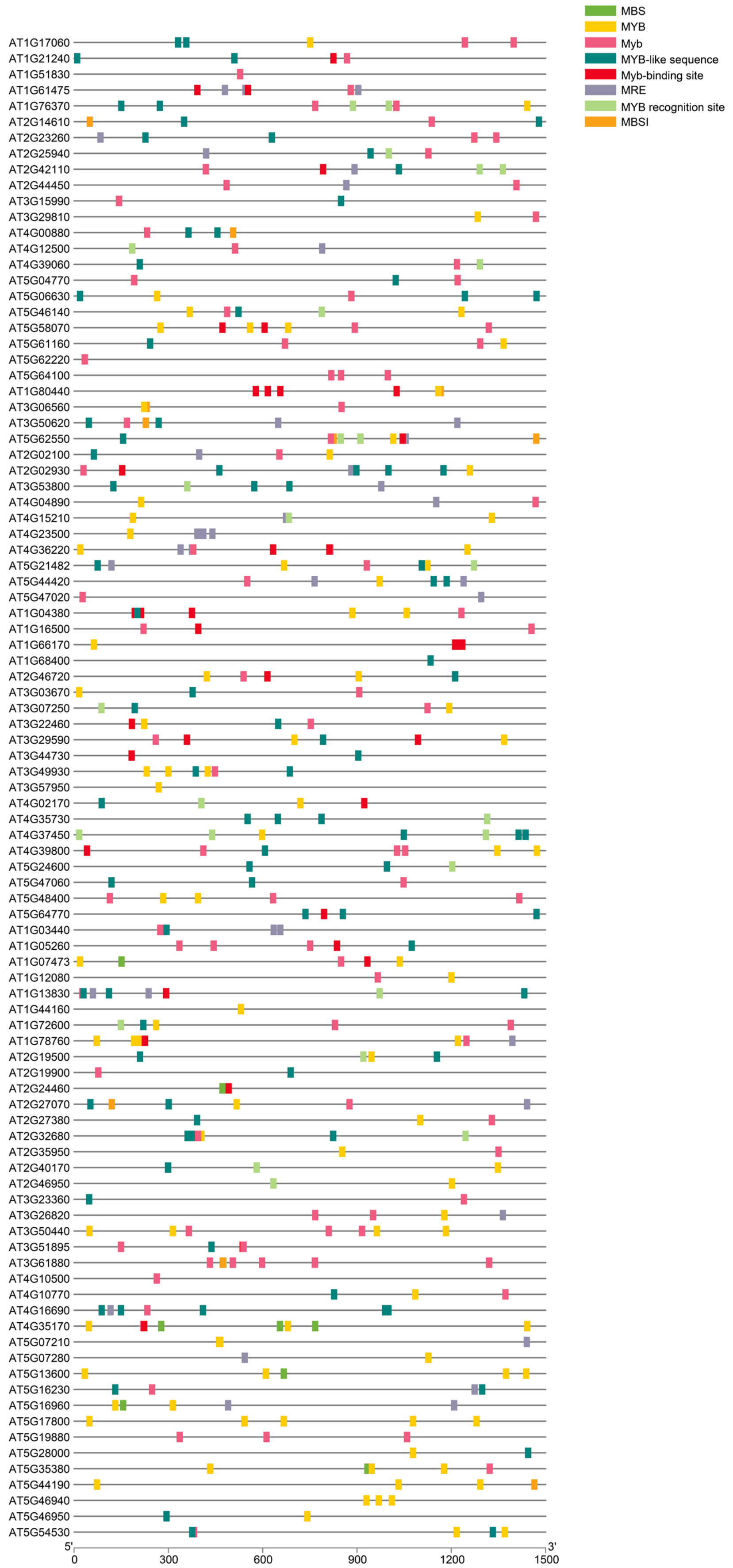

Figure S4. Putative MYB Binding sites in the 1.5-kb promoter region.

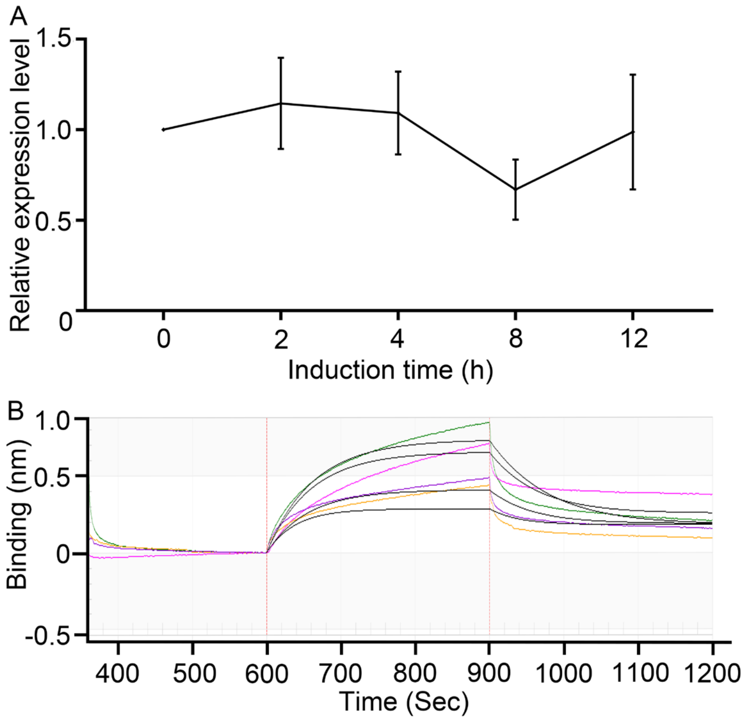

Figure S5. PGA37 was unable to activate the expression of *GLK1*. **a** qRT-PCR analysis of *GLK1* expression in *35S*::*PGA37-GR* transgenic seedlings treated with 10 μM DEX for various times. **b** Binding curves of PGA37 with *GLK1* promoter.

Table S1 Putative PGA37 targets identified by RNA-Seq

GeneID	log2 Ratio (DEX/WT)	P-value	FDR	Description
AT3G23360	13.97	0	0	Protein phosphatase 2C family protein
AT5G46950	13.09	2.07E-09	2.66E-07	Plant invertase/pectin methylesterase inhibitor superfamily protein
AT5G46940	12.22	8.13E-06	0.00051	Plant invertase/pectin methylesterase inhibitor superfamily protein
AT2G24460	11.67	2.05E-06	0.00015	BEST Arabidopsis thaliana protein match is: Zinc finger, C3HC4 type (RING finger) family protein
AT2G35950	11.36	6.44E-05	0.00339	EDA12 | embryo sac development arrest 12
AT5G07210	11.25	3.27E-08	3.44E-06	ARR21, RR21 | response regulator 21
AT2G27070	10.62	3.23E-05	0.00179	ARR13, RR13 | response regulator 13
AT5G17800	4.62	1.56E-06	0.00012	AtMYB56, MYB56 | myb domain protein 56
AT2G19500	4.56	2.71E-05	0.00154	CKX2, ATCKX2 | cytokinin oxidase 2
AT1G44160	4.16	1.58E-05	0.00095	HSP40/DnaJ peptide-binding protein
AT1G12080	4.08	4.61E-07	4.05E-05	Vacuolar calcium-binding protein-related
AT5G44190	4.05	7.28E-05	0.00374	GLK2, ATGLK2, GPRI2 | GOLDEN2-like 2
AT5G28000	3.97	2.71E-05	0.00153	Polyketide cyclase/dehydrase and lipid transport superfamily protein
AT3G50440	3.91	9.05E-06	0.00056	ATMES10, MES10 | methyl esterase 10
AT2G40170	3.48	2.71E-05	0.00153	ATEM6, GEA6, EM6 | Stress induced protein
AT3G26820	3.11	8.20E-05	0.00417	Esterase/lipase/thioesterase family protein
AT2G32680	3.09	2.01E-08	2.23E-06	AtRLP23, RLP23 | receptor like protein 23
AT2G19900	3.08	8.50E-07	6.94E-05	ATNADP-ME1, NADP-ME1 | NADP-malic enzyme 1
AT2G27380	3.01	8.50E-07	6.96E-05	ATEPR1, EPR1 | extensin proline-rich 1
AT5G16230	2.92	5.60E-14	1.67E-11	Plant stearoyl-acyl-carrier-protein desaturase family protein
AT5G19880	2.91	8.33E-14	2.35E-11	Peroxidase superfamily protein
AT4G35170	2.88	2.74E-05	0.00154	Late embryogenesis abundant (LEA) hydroxyproline-rich glycoprotein family

AT3G61880	2.88	2.74E-05	0.00154	CYP78A9 | cytochrome p450 78a9
AT4G10770	2.73	1.60E-05	0.00095	ATOPT7, OPT7 | oligopeptide transporter 7
AT5G13600	2.64	1.33E-07	1.24E-05	Phototropic-responsive NPH3 family protein
AT5G16960	2.57	4.13E-05	0.00226	Zinc-binding dehydrogenase family protein
AT3G51895	2.43	1.17E-13	3.25E-11	SULTR3;1, AST12 | sulfate transporter 3;1
AT1G05260	2.42	1.56E-12	3.55E-10	RCI3, RCI3A | Peroxidase superfamily protein
AT5G54530	2.40	1.58E-06	0.00012	protein of unknown function, DUF538
AT4G16690	2.34	8.65E-07	7.04E-05	ATMES16, MES16 | methyl esterase 16
AT4G10500	2.28	2.01E-08	2.22E-06	2-oxoglutarate (2OG) and Fe(II)-dependent oxygenase superfamily protein
AT1G07473	2.15	8.22E-10	1.12E-07	hypothetical protein
AT1G72600	2.14	8.60E-05	0.00435	hydroxyproline-rich glycoprotein family protein
AT2G46950	2.13	3.89E-06	0.00027	CYP709B2 | cytochrome P450, family 709, subfamily B, polypeptide 2
AT5G35380	2.11	0	0	Protein kinase protein with adenine nucleotide alpha hydrolases-like domain
AT1G03440	2.09	1.58E-09	2.06E-07	Leucine-rich repeat (LRR) family protein
AT2G13910	2.08	3.32E-11	5.54E-09	pseudogene, CHP-rich zinc finger protein
AT1G13830	2.01	3.50E-09	4.37E-07	Carbohydrate-binding X8 domain superfamily protein
AT1G78760	1.98	1.18E-13	3.22E-11	F-box/RNI-like superfamily protein
AT5G07280	1.78	2.19E-05	0.00127	EMS1, EXS | Leucine-rich repeat transmembrane protein kinase
AT3G07250	1.74	0	0	nuclear transport factor 2 (NTF2) family protein / RNA recognition motif (RRM)-containing protein
AT5G44420	1.71	7.02E-10	9.70E-08	PDF1.2, PDF1.2A, LCR77 | plant defensin 1.2
AT1G66170	1.65	1.78E-15	6.17E-13	MMD1 | RING/FYVE/PHD zinc finger superfamily protein
AT3G22460	1.64	2.75E-10	3.97E-08	OASA2 | O-acetylserine (thiol) lyase (OAS-TL) isoform A2

AT1G17060	1.62	0	0	CYP72C1, SOB7 | cytochrome p450 72c1
AT3G49930	1.62	9.71E-07	7.73E-05	C2H2 and C2HC zinc fingers superfamily protein
AT2G42110	1.57	3.83E-06	0.00027	hypothetical protein
AT1G76370	1.52	9.57E-14	2.67E-11	Protein kinase superfamily protein
AT4G23500	1.48	4.65E-10	6.57E-08	Pectin lyase-like superfamily protein
AT2G02930	1.43	1.89E-11	3.43E-09	ATGSTF3, GST16, GSTF3 | glutathione S-transferase F3
AT2G46720	1.43	1.17E-07	1.10E-05	HIC, KCS13 | 3-ketoacyl-CoA synthase 13
AT3G29590	1.43	4.30E-05	0.00234	AT5MAT | HXXXD-type acyl-transferase family protein
AT5G57480	1.43	0	0	P-loop containing nucleoside triphosphate hydrolases superfamily protein
AT3G06560	1.43	6.62E-07	5.54E-05	PAPS3 | poly(A) polymerase 3
AT1G04380	1.40	0	0	2-oxoglutarate (2OG) and Fe(II)-dependent oxygenase superfamily protein
AT3G03670	1.37	6.98E-06	0.00045	Peroxidase superfamily protein
AT2G25940	1.36	0	0	ALPHA-VPE, ALPHAVPE | alpha-vacuolar processing enzyme
AT5G58070	1.35	0	0	ATTIL, TIL | temperature-induced lipocalin
AT4G37450	1.28	0	0	AGP18, ATAGP18 | arabinogalactan protein 18
AT4G02170	1.28	0	0	unknown protein
AT3G53800	1.27	2.44E-06	0.00017	Fes1B | Fes1B
AT5G06630	1.27	8.80E-05	0.00444	proline-rich extensin-like family protein
AT5G47020	1.26	2.16E-13	5.62E-11	unknown protein
AT1G16500	1.26	8.56E-08	8.30E-06	filamentous hemagglutinin transporter
AT5G47060	1.25	1.27E-14	3.97E-12	hypothetical protein
AT1G61475	1.24	4.95E-06	0.00033	ATP binding;protein kinases
AT5G61160	1.22	1.72E-13	4.65E-11	AACT1 | anthocyanin 5-aromatic acyltransferase 1
AT1G68400	1.21	0	0	leucine-rich repeat transmembrane protein kinase family protein
AT3G50620	1.18	1.18E-05	0.00072	P-loop containing nucleoside triphosphate hydrolases superfamily protein
AT2G44450	1.18	3.94E-13	9.74E-11	BGLU15 | beta glucosidase 15

AT4G39800	1.17	1.74E-13	4.66E-11	MI-1-P SYNTHASE, MIPS1, ATMIPS1, ATIPS1 | myo-inositol-1-phosphate synthase 1
AT5G64100	1.17	0	0	Peroxidase superfamily protein
AT5G04770	1.14	3.84E-05	0.00212	ATCAT6, CAT6 | cationic amino acid transporter 6
AT2G02100	1.14	1.17E-05	0.00072	LCR69, PDF2.2 | low-molecular-weight cysteine-rich 69
AT2G14610	1.14	3.89E-08	3.98E-06	PR1, PR 1, ATPR1 | pathogenesis-related gene 1
AT3G44730	1.13	5.21E-13	1.25E-10	ATKP1, KP1 | kinesin-like protein 1
AT4G35730	1.12	2.66E-15	9.02E-13	Regulator of Vps4 activity in the MVB pathway protein
AT4G36220	1.11	5.28E-08	5.27E-06	FAH1, CYP84A1 | ferulic acid 5-hydroxylase 1
AT5G62550	1.11	8.80E-05	0.00444	microtubule-associated futsch-like protein
AT4G04890	1.10	4.10E-06	0.00028	PDF2 | protodermal factor 2
AT5G24600	1.10	1.59E-05	0.00095	TRP-like ion channel protein
AT3G29810	1.09	2.21E-08	2.42E-06	COBL2 | COBRA-like protein 2 precursor
AT1G80440	1.08	2.19E-05	0.00127	Galactose oxidase/kelch repeat superfamily protein
AT5G21482	1.08	7.19E-05	0.00371	CKX7, ATCKX5 | cytokinin oxidase 7
AT4G39060	1.06	9.75E-07	7.74E-05	BEST Arabidopsis thaliana protein match is: Galactose oxidase/kelch repeat superfamily protein
AT4G15210	1.05	4.11E-05	0.00225	ATBETA-AMY, AT-BETA-AMY, RAM1, BMY1, BAM5 | beta-amylase 5
AT2G23260	1.05	2.05E-05	0.0012	UGT84B1 | UDP-glucosyl transferase 84B1
AT1G21240	1.05	1.12E-06	8.68E-05	WAK3 | wall associated kinase 3
AT5G62220	1.03	2.56E-11	4.42E-09	ATGT18, GT18 | glycosyltransferase 18
AT3G15990	1.01	0	0	SULTR3;4 | sulfate transporter 3;4
AT1G51830	-1.05	9.23E-09	1.08E-06	Leucine-rich repeat protein kinase family protein
AT4G00880	-1.09	1.74E-05	0.00103	SAUR-like auxin-responsive protein family
AT5G46140	-1.18	8.12E-06	0.00051	Protein of unknown function (DUF295)
AT5G48400	-1.21	1.43E-06	0.00011	ATGLR1.2, GLR1.2 | Glutamate receptor family protein

AT5G64770	-1.28	5.60E-05	0.00299	RGF9 | Encodes a root meristem growth factor (RGF). Belongs to a family of functionally redundant homologous peptides that are secreted, tyrosine-sulfated, and expressed mainly in the stem cell area and the innermost layer of central columella cells. RGFs are required for maintenance of the root stem cell niche and transit amplifying cell proliferation.
AT3G57950	-1.49	4.20E-06	0.00029	unknown protein
AT4G12500	-1.61	2.03E-07	1.84E-05	Bifunctional inhibitor/lipid-transfer protein/seed storage 2S albumin superfamily protein

Table S2 Primers used in this study

Primer	Sequence (5' to 3')	Experiment
GRF	tcccccgggactagtATTCAGCAAGCCACTGC AGGAGT	Construction of the pBA-HA-GR vector
GRB	gctctagaAGCTAGCTTACTCAGTTAGGTC	Construction of the HA-GR-pBA vector
PGA37GRF	ggggtaccggcgcgccATCAATTACTTTCCTCG TTATCC	Construction of the pBA-35S::PGA37-GR vector
PGA37GRB	ggactagtAAGACGACCATGAGCAATCATC	Construction of the pBA-35S::PGA37-GR vector
ACT7F	GGAACTGGAATGGTGAAGGCTG	Internal reference for RT-PCR
ACT7B	CGATTGGATACTTCAGAGTGAGGA	Internal reference for RT-PCR
PGA37RTF	CCTCACACATATGAAATACCATC	Genotyping of *myb118* and RT-PCR of *PGA37*
PGA37RTB	CCTCTTCTTCAGTCCATCCATC	Genotyping of *myb118* and RT-PCR of *PGA37*
LEC1RTF	CTCCCTTCTCTCACTATCAG	RT-PCR of *LEC1*
LEC1RTR	GTCGTCAGAGATTTTGGCGTG	RT-qPCR of *LEC1*
ACT7qRTF	GGAACTGGAATGGTGAAGGCTG	Internal reference for RT-qPCR
ACT7qRTB	CGATTGGATACTTCAGAGTGAGGA	Internal reference for RT-qPCR
HY5qRTF	AAGCTGTCGGAAAAGAAACTTC	RT-qPCR of *HY5*
HY5qRTB	GAAACTCTGTTCCTCAACAACC	RT-qPCR of *GLK1*
GLK1qRTF	AAGGGAAGAGAAAGGTGAAGGTG	RT-qPCR of *GLK1*
GLK1qRTB	GTTGTGACGAGTGAGACAATGGA	RT-qPCR of *GLK1*
GLK2qRTF	TAGCGGAAGAGATGAGGAACAAG	RT-qPCR of *GLK2*

GLK2qRTB	TAAAACCACCGTCACCACCAC	RT-qPCR of *GLK2*
ARR13qRTF	GTCGTGCATCAAGGTAGGTTTAG	RT-qPCR of *ARR13*
ARR13qRTB	TCACGTGCTTCGGACAAGAGTC	RT-qPCR of *ARR13*
ARR21qRTF	ATTCAGTGGACGGATTCTCTTCAC	RT-qPCR of *ARR21*
ARR21qRTB	AACCCTGCTCCGCAACTCTTC	RT-qPCR of *ARR21*
